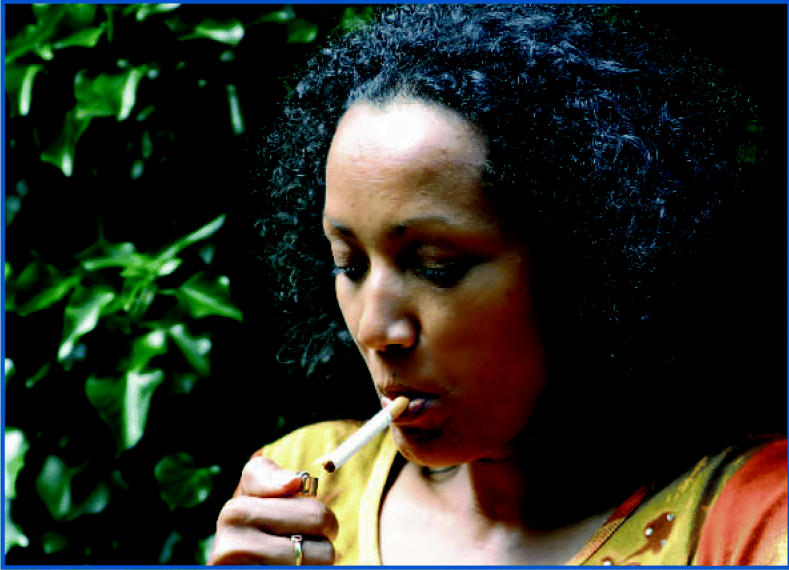# Headliners: Genetic Research: Polymorphisms Modify Breast Cancer Risk in Smokers

**Published:** 2006-11

**Authors:** Jerry Phelps

Mechanic LE, Millikan RC, Player J, de Cotret AR, Winkel S, Worley K, et al. 2006. Polymorphisms in nucleotide excision repair genes, smoking and breast cancer in African Americans and whites: a population-based case–control study. Carcinogenesis 27:1377–1385.

Previous research has established cigarette smoking as a risk factor for a number of cancers, including those of the lung, pancreas, and head and neck. A link between cigarette smoking and breast cancer is not as clear; however, scientists do know that breast cancer occurs at different rates in different racial groups. In this paper, NIEHS grantee Robert C. Millikan of the University of North Carolina at Chapel Hill and his colleagues document findings suggesting that specific combinations of polymorphisms on certain nucleotide excision repair (NER) genes may modify the risk of breast cancer in black women who smoke.

NER is the primary means by which smoking-induced DNA damage is repaired. There are several known polymorphisms on genes involved in NER. These investigators conducted a genetic epidemiologic study aimed at determining whether such polymorphisms alter the association between smoking and breast cancer.

The investigators analyzed exposure histories and DNA samples extracted from peripheral blood lymphocytes of 3,863 women (1,449 black, 2,414 white) aged 21 to 74. The women were participating in the Carolina Breast Cancer Study, a large population-based case–control study of breast cancer in North Carolina. Next the investigators calculated odds ratios for breast cancer and smoking and for breast cancer and nine polymorphisms on six NER genes. Then they examined how odds ratios for smoking were modified by single and combined NER genotypes.

In general, they found, smoking was more strongly associated with breast cancer in black women than in white women. The association increased even more for black women with particular patterns of polymorphisms when combined with different smoking characteristics including amount of smoking, duration, time since smoking cessation, age at smoking initiation, and being a former smoker. No interactions were seen in white women.

The investigators believe this is the first study to examine multiple NER polymorphisms together as susceptibility factors for breast cancer in combination with smoking. Further studies with larger numbers of participants are needed to confirm these results.

## Figures and Tables

**Figure:**